# Examining the association between HIV prevalence and socioeconomic factors among young people in Zambia: Do neighbourhood contextual effects play a role?

**DOI:** 10.1371/journal.pone.0268983

**Published:** 2022-06-08

**Authors:** Chola Nakazwe, Knut Fylkesnes, Charles Michelo, Ingvild F. Sandøy

**Affiliations:** 1 Department of Global Public Health and Primary Care, Centre for International Health, University of Bergen, Bergen, Norway; 2 School of Public Health, University of Zambia, Lusaka, Zambia; 3 Zambia Statistics Agency, Lusaka, Zambia; 4 Centre for Intervention Science in Maternal and Child Health, University of Bergen, Bergen, Norway; University of Salamanca, SPAIN

## Abstract

**Background:**

The study examined the association between HIV infection and individual and neighbourhood-level socioeconomic factors in Zambia.

**Methods:**

We used multilevel mixed effects logistic regression to examine the association of individual and neighbourhood level variables on HIV prevalence based on data from the 2013–14 and 2018 Zambia Demographic and Health Surveys, population-based cross-sectional surveys. The analysis was restricted to young people (15–24 years) with HIV serostatus results (n = 11,751 and n = 10,154). HIV serostatus was the outcome variable and socioeconomic status was measured by wealth, education and employment.

**Results:**

Overall, at individual level, education was associated with reduced odds of HIV infection among young women and men. Conversely, relative wealth was generally associated with increased odds of infection for both young women and men. Young, employed men were at reduced odds of HIV infection than the unemployed. Living in neighbourhoods with higher average level of education was associated with higher odds of HIV infection. In 2013–14, 13% and 11% of the variation in HIV infection among young men and women was attributed to neighbourhoods, while 20% and 11% variation was attributed to neighbourhoods in 2018. Inclusion of individual and neighbourhood variables in the full regression model accounted for 65.7% and 59.5% of explained variance in 2013–14 and 64.6% and 44.3% in 2018, for women and men, respectively. This reduced unexplained variance by an average of 56% in 2013–14 and 29% in 2018.

**Conclusion:**

We found that HIV infection among young people in Zambia is more strongly associated with individual-level socioeconomic factors compared to neighbourhood factors. Individual-level education remains an important socioeconomic factor associated with reduced odds of HIV infection. This suggests that the HIV response in Zambia should still focus on individual level prevention strategies.

## Introduction

Globally the HIV epidemic differs by region in terms of prevalence levels and patterns, with a high HIV morbidity and mortality burden in sub-Saharan Africa (SSA) in particular. In 2017, 66% of the 1.8 million new HIV infections estimated globally occurred in this region, and AIDS-related deaths in SSA were estimated at 660,000 [1]. The risk of HIV infection is associated with a number of behavioural risk factors including but not limited to multiple and concurrent sexual partnerships, which again, are linked to individual demographic characteristics (such as age, sex, and marital status) and socioeconomic factors (such as education, wealth, and employment) [2].

The associations between HIV prevalence and socioeconomic factors have changed over time in SSA as sexual behaviours have changed for different socioeconomic groups [3,4]. In Zambia for instance, premarital sex declined for both females and males by 17% between 2000 and 2009. The decline in premarital sex among young women was particularly marked for those with low education at individual level, rural residents, and those residing in neighbourhoods with high educational attainment. Among young men significant declines in premarital sex were seen irrespective of level of individual educational attainment and rural/urban residence, and those residing in neighbourhoods of medium educational attainment and medium or high labour force participation. There was also a substantial decline in multiple sexual partnerships among males, while among females, multiple sexual partnerships remained stable, over the same period [3]. Among young men declines in multiple sexual partnerships were associated with almost all socio-economic factors; individual educational attainment, rural and urban residence, and those residing in neighbourhoods with medium educational attainment. Understanding how these factors influence HIV infection at different times and in different settings can help public health authorities and planners design targeted and comprehensive prevention strategies.

Available evidence indicates that individual-level socioeconomic factors are strongly associated with HIV infection, though the association is complex and depends on sex, urban/rural residence, and the phase of the epidemic (i.e. how the HIV epidemic has evolved over time). Relative wealth appears to have a mixed influence on HIV risk: Mishra et al., examined the association between HIV prevalence and wealth across eight sub-Saharan countries using data from studies conducted between 2003 to 2005 and found that wealthier individuals were more likely to be HIV positive [5]. Msisha et al., also observed that low relative wealth was associated with lower odds of being HIV positive in Tanzania in study conducted in 2003–4 [6]. Exploring the connections between HIV serostatus and individual, household, and community socioeconomic resources in Kenya, Ishida et al. found that the burden of HIV shifted from higher to lower socioeconomic status (SES) subgroups between 2003 and 2007 [7], highlighting the fact that the relationship between SES and HIV is context- and time-specific. Similar findings of an association between low wealth status and likelihood of HIV positivity were observed in other studies conducted in Zimbabwe, South Africa and other sub-Saharan African countries [8–10]. In a 2013 study, Magadi’s analysis of data from 20 SSA countries conducted between 2003 to 2008, revealed that urban poor in SSA had significantly higher odds of HIV infection than their non-poor counterparts [8]. Lopman et al., in a 1998 to 2003 population-based open cohort study in Eastern Zimbabwe, also found that HIV incidence was lower in the higher wealth tercile [9]. Likewise, a 2003 to 2005 longitudinal population-based study in rural South Africa by Bärnighausen et al. revealed that individuals living in households with middle-level relative wealth were at the highest risk of HIV acquisition [10]. The mixed findings may be attributed to the phase of the epidemic and to contextual and neighbourhood-level factors. According to Gillespie et al., accounting for a number of underlying contextual factors such as urban/rural residence, community wealth and mobility, reduced the positive association between wealth status and HIV based on findings of a review of studies conducted between 2004 and 2007 in sub-Saharan African countries with high HIV prevalence [11].

The association between employment and HIV prevalence also shows inconsistent patterns. Msisha et al., investigating socioeconomic status and HIV seroprevalence in Tanzania based on a study conducted in 2003–4, found that employment status was positively associated with high HIV prevalence. Men and women employed in professional (i.e., non-agricultural or manual-related) occupations were at a higher risk of infection compared to those in the agricultural sector. However, for men the association changed after adjusting for other covariates, with unemployed men having a three-fold higher risk of being infected compared to those in the agricultural sector [12]. Conversely, the association remained the same for females after adjusting for other covariates, with professional women associated with high HIV prevalence. Bunyasi and Coetzee found no significant association between employment and HIV prevalence in their study based on the 2007 and 2008 Prevention of Mother to Child Transmission of HIV, Effectiveness in Africa and Research Linkages to HIV Care survey (PEARL study) in South Africa [13].

Education in general appears to be protective with regard to HIV risk [10,14–16]. Barnighausen et al. in a 2003 to 2005 study found that educational attainment significantly reduced the hazard of becoming infected in a rural community in South Africa [10]. Investigating change over time in HIV prevalence by educational attainment in selected urban and rural communities in Zambia, Michelo et al. found that there was a shift towards reduced risk of HIV infection in subgroups with higher education from 1995 and 2003 [15]. The dominating patterns in many SSA countries is that the association has transitioned from a higher risk of HIV infection among the educated, in the early phase of the epidemic, to a lower risk of infection among the more highly educated as the epidemic matures. According to Hargreaves et al., studies conducted from 1996 onwards were more likely to find a lower risk of HIV infection among the most educated [16].

Most studies on factors associated with HIV infection have primarily focused on individual-level characteristics, but neighbourhood-level factors can also be independently associated with HIV infection [11]. A systematic review by Peterson et al., evaluating the influence of contextual factors on HIV/AIDS, sexually transmitted infections, and risky sexual behaviour in sub-Saharan Africa, based on studies conducted from 1995 to 2017, showed contextual factors to be significantly associated with HIV infection [17]. Three of the included studies found associations between neighbourhood-level poverty and likelihood of HIV positivity, of these, one found that women living in communities with lower socioeconomic status (SES) had higher risk of HIV infection. In contrast, some studies found lower likelihood of HIV infection in poorer communities [17]. Two studies conducted among young women in selected communities in Zambia between 1997–1998 and 2003 found that neighbourhood-level factors were as strongly associated with HIV infection as individual factors [14,18]. A similar analysis of both individual and neighbourhood level socioeconomic factors in relation to HIV risk has not yet been done at national level in Zambia. Our study extends previous research by using nationally representative data from the 2013–14 and 2018 Zambia Demographic and Health Survey (ZDHSs) and applying multilevel analysis to investigate the association between HIV infection and individual and neighbourhood-level wealth, employment, and education among young women and men two decades after the peak of the epidemic.

## Methods

### Data source and study population

We analysed data from the 2013–14 and 2018 Zambia Demographic and Health Survey (ZDHS). Population-based cross-sectional surveys with a two-stage stratified cluster sample design. The eligible study population included women aged 15–49 years and men aged 15–59 years who were either permanent residents of the sampled households or visitors present in the households on the night before the survey [19,20]. In, 2013–14, a total of 16,411 women and 14,773 men were successfully interviewed, yielding response rates of 96.2% and 91.1%, respectively. Similarly in 2018, 13,683 women and 12,132 men were successfully interviewed, yielding response rates of 96.4% and 91.6%, respectively. The principal reason for non-response among eligible adults was failure to find individuals at home despite repeated visits, followed by refusal to be interviewed. In 2013–14, of the respondents who completed the structured interview, 29,007 respondents aged 15–59 years were tested for HIV, corresponding to a response rate of 87.2%. Young people aged 15–24 years accounted for 39.9% of the people tested or 11,571 in total. Further details about participation in the survey and HIV testing response rates can be found in the 2013–14 ZDHS report [19]. In 2018, 91% of respondents tested for HIV (24,702), with young people accounting for 41.1% of the people tested or a total of 10,154. Further details about participation in the survey and HIV testing response rates can be found in the 2018 ZDHS report [20].

The 2013–14 ZDHS HIV testing protocol allowed for linking of the HIV results to the socio-demographic data. The HIV Laboratory testing algorithm used for the DHS surveys followed the 2005 UNAIDS/WHO guidelines for HIV testing in population based surveys. Eligible women and men who consented to HIV testing were asked to voluntarily provide about five drops of blood from a finger prick for anonymous testing. Each Dried Blood Spots (DBS) sample was given a bar code label, and a duplicate label was attached to the individual’s questionnaire, while the third bar code was attached to the blood sample transmittal form to track the blood sample from the field to the central laboratory [19]. This testing algorithm included Vironostika HIV antigen/antibody combination assay (Biomerieux) as the first assay and Enzygnost HIV Integral II assay (Dade Behring) as the second confirmatory testing assay. Both tests are Fourth-generation enzyme-linked immunoassay assays (ELISAs) [21]. Western Blot was used as a third confirmatory test. Further details about the testing methodology can be found in the 2013–14 ZDHS report and in an article we published, which is based on the same data [19,22].

For respondents who wanted to know their HIV status, home-based counselling and testing were offered in parallel following the national HIV testing algorithm to ascertain HIV infection status. Respondents were tested for HIV using Determine^™^ HIV ½ (Alere Healthcare) and Uni-Gold^™^ (Trinity Biotechnology) rapid diagnostic tests, which were run concurrently.

In 2015 UNAIDS/WHO guidelines for HIV testing in population based surveys were revised taking into account concerns raised that enzyme-immunoassay (EIA) testing strategies may result in overestimation of HIV prevalence. The new guidelines recommend the use of a more specific confirmatory assay to assess all enzyme immunoassay-reactive specimens [23]. The 2013/14 ZDHS blood specimens were retested in 2017 following these new guidelines to assess the extent of overestimation and to obtain a confirmed HIV prevalence estimate. Confirmed refers to an estimate verified with a more specific assay. DBS blood specimens from respondents classified as HIV positive according to the original survey laboratory testing algorithm and who had one or two negative home based Rapid Diagnostic Tests (RDT) conducted in the field, or no RDT results at all, were tested using Inno–Lia HIV I/II assay. The results of the Inno-Lia assay were taken as the final confirmatory result. Blood specimens from laboratory positive respondents with a positive RDT result were considered confirmed positive with no Inno-Lia testing [24].

The 2018 ZDHS HIV testing protocol also allowed for linking of the HIV results to the socio-demographic data. The testing algorithm for this survey included two Enzyme immunoassays (EIAs) namely, Bioelisa HIV-1+2 Ag/Ab assay (Biokit, Spain) as the first assay and Genscreen ULTRA Ag/Ab (Bio-rad, France) as the second confirmatory testing assay. The Geenius HIV 1/2 supplementary assay was used as a confirmatory test of all double EIA positive samples. Further details about the testing methodology can be found in the 2018 ZDHS report [20].

### Variable definition

The selection of variables to include in the analysis of this article was based on the proximate determinants framework for factors affecting the risk of transmission of HIV developed by Boerma and Weir [25]. According to this conceptual framework underlying variables, such as socioeconomic status influence proximate determinants, which in turn have an effect on biological mechanisms to influence health outcomes (i.e. HIV infection). The proximate determinants include among others concurrent sexual partnerships, number of sexual partners and condom use. The conceptual framework helps to understand the causal pathways from distal socioeconomic factors to HIV infection The focus was on underlying determinants, i.e., the socioeconomic context of the neighbourhood (neighbourhood educational attainment, wealth, and employment status) and individual demographic and socioeconomic factors. The operational definitions are as follows.

#### Outcome variable

The dependent variable for this study was HIV status, which is defined as serostatus determined by testing blood samples collected from each consenting individual (0 indicating “HIV negative” and 1 “HIV positive”).

#### Independent variables

*Individual level*. The main variables of interest were educational attainment, wealth, and employment status. The **wealth index score** from the ZDHS was used, and separate wealth tertiles for urban and rural populations were created to reduce residence bias. The wealth score was calculated from the first component of principal component analysis (PCA) of household assets, housing characteristics, and access-to-amenities data (e.g., roof and floor material, electricity, water supply, possession of goods such as a bicycle and television, and so forth). The methodology used to calculate the wealth index is based on the Filmer and Pritchett approach and a detailed explanation is available in the Measure DHS report [26]. Educational attainment was defined as the reported number of years spent in school, and this was used as a continuous variable in the regression model. Employment status was measured by asking respondents if they had been working in the 12 months preceding the survey and this was then categorised as unemployed and employed. Occupation was defined as the main type of work done in the 12 months preceding the survey, and this was classified using the ILO International Standard Classification of Occupation Codes (ISCO). However, this variable was not included in the regression model, as it partly represents the same information as employment status. In addition, we included sex, age, marital status and residence in the regression models to adjust for potential confounding.

*Neighbourhood level*. In this study, enumeration areas were used as a proxy for neighbourhoods. An enumeration area is the smallest geographic area used in census of populations and other types of surveys, with an average size of 130 households or 600 people. People residing in these areas usually have similar characteristics. Variables describing the characteristics of the neighbourhoods were derived by aggregating individual responses within each enumeration area (cluster) for all respondents and then categorising the means or proportions into three levels (low, medium, and high). Neighbourhood educational attainment was derived by calculating the mean number of years of school attained by the individuals who were interviewed in each enumeration area. Neighbourhood employment was the proportion of individuals interviewed in each enumeration area who were categorised as employed in the last 12 months. Neighbourhood relative wealth was derived by calculating the mean of the wealth scores of all respondents in the enumeration area.

### Analysis

We restricted the analyses to young people (15–24 years). We conducted the analyses of both sexes and then stratified by women and men. Analyses were carried out in two phases. In the first phase we explored bivariate associations between HIV serostatus and individual demographic and socioeconomic factors, and neighbourhood-level socioeconomic factors. In the second phase, we used multilevel mixed-effects logistic regression analyses on HIV status with associated factors, which we conducted using Stata 15. Multilevel mixed effects logistic regression takes into account both individual and neighbourhood-level variability. The bivariate and multivariate analyses also controlled for the potential confounder age, which was adjusted for as a linear effect. Collinearity was assessed using the variance inflation factor, and after concluding that there was limited collinearity, all the demographic and socioeconomic variables explored in the bivariate analysis were included in the multivariate analysis. We tested five separate models to examine the association between HIV prevalence and socioeconomic factors. Model 1 was the null or random intercept-only model, which did not include any socioeconomic or demographic factors. Subsequent models gradually included socioeconomic and demographic variables, starting with individual-level variables only, then neighbourhood-level variables only, and finally included both individual and neighbourhood-level variables (corresponding to model 2, model 3 and model 4, respectively). The final model included only significant variables at both individual and neighbourhood level. The likelihood-ratio test was used for assessing the goodness-of-fit of the models and to determine whether adding independent variables to the intercept-only model significantly improved the fit.

Multilevel statistics estimated were, explained variance, the interclass correlation (ICC)), and log likelihood tests. To estimate explained variance (R^2^), we use the approach explained in Hox, J (2010), and proposed by Snijders and Bosker (1999) for multilevel mixed effects logistic regression models, which does not rely on the likelihood. The estimated variance is decomposed into the lowest-level residual variance ($\sigma_{R}^{2}$), which is fixed to π^2^/3 = 3.29 in logistic models, the second-level variance ($\tau_{0}^{2}$) and the variance of the linear predictor from the fixed part of the model ($\sigma_{F}^{2}$) [27]. The explained variance is estimated using the formula:

$$R^{2}=\frac{\sigma_{F}^{2}}{\sigma_{F}^{2}+\tau_{0}^{2}+\sigma_{R}^{2}}$$

Where:

$\sigma_{F}^{2}$ is the explained part of the total variance

$\tau_{0}^{2}$ is the unexplained variance at the neighbourhood level

$\sigma_{R}^{2}$ is the unexplained variance at the individual level (assumed to be π^2^/3 = 3.29).

The ICC or rho indicates the proportion of the variance explained by the grouping structure in the population, which in our case are the neighbourhoods [27]. This is same as the unexplained neighbourhood-level variance as a proportion of total variance. We tested for interactions in the final model, for individual and neighbourhood variables, but none were found to be statistically significant.

### Ethics

Ethical approval for the 2013–14 ZDHS was obtained from the Tropical Disease and Research Centre (TDRC) ethical committee, the institutional board of ICF International, and the Centers for Disease Control and Prevention (CDC) Atlanta research ethics review board. Ethical approval for the 2018 ZDHS was obtained from the Tropical Disease and Research Centre (TDRC) ethical committee and the institutional board of ICF International. In both the 2013–14 and 2018 surveys, participation in the surveys was obtained by soliciting verbal informed and voluntary consent. Participants were informed that the survey’s HIV testing results were anonymous. Home-based counselling and testing were offered in parallel to participants following the national HIV testing algorithm to ascertain HIV infection status for respondents who consented and wanted to know their HIV status. Concurrent HIV testing with Determine^™^ HIV ½ (Alere Healthcare) and Uni-Gold^™^ (Trinity Biotechnology) was the home-based testing procedure, and nurses and lay counsellors provided pre- and post-test counselling. If either of the rapid tests was HIV reactive (positive), the respondent was referred to the nearest health facility for further assessment, treatment, and care.

## Results

### Demographic characteristics of the study participants

In total, 11,571 young people aged 15–24 years were interviewed and consented to HIV testing in 2013–14, while 10,154 were interviewed and tested in 2018. More than fifty percent were living in rural areas, during the two study years. There were more young women than men in the study population (52% and 53%, compared to 48% and 47% in both 2013–14 and 2018) (see S1 & S2 Tables).

### HIV prevalence and associated factors

An estimated 4.5% of the young people were HIV positive in 2013–14 and this declined to 3.8% in 2018. The prevalence was notably higher among young people residing in urban areas than in rural areas (6.6% and 5.3% compared with 2.4% and 2.6%, respectively). Age was associated with higher odds of being infected. Young people aged 20–24 were more likely to be infected than those in the younger age group of 15–19 years. Formerly married young people were at increased odds of being infected (aOR 3.25, 95% CI 2.26–4.67 and aOR 4.03,95% CI 2.66–6.10) compared to the never-married (formerly married include those who were separated, divorced and widowed). Conversely, married women had lower odds of being infected (aOR 0.80 95% CI 0. 63–1.03) and (aOR 0.82 95% CI 0.61–1.11) (Table 2).

HIV prevalence and age-adjusted odds ratios varied by the selected socioeconomic factors. Education was associated with reduced odds of infection, particularly for young men in both 2013–14 and 2018. Among both young men and women, those with the highest wealth status had higher odds of being infected (aOR 2.33, 95% CI 1.36–3.99 for men, and aOR 2.70, 95% CI 2.00–3.65 for women in 2013–14) and (aOR 2.51, 95% CI 1.29–4.88 for men, and aOR 1.79, 95% CI 1.25–2.54 for women in 2018) than those in the poorest tertile (Table 2). Employment was associated with lower odds of being infected (aOR 0.67, 95% CI 0.55–0.80) and (aOR 0.75, 95% CI 0.60–0.95) and compared to those unemployed (Table 1). The agricultural occupation was associated with lower odds of HIV infection among young men and women in both years ((aOR 0.27, 95% CI 0.15–0.49 and aOR 0.45, 95% CI 0.32–0.63) in 2013–14 and ((aOR 0.55, 95% CI 0.25–1.22 and aOR 0.67, 95% CI 0.44–1.03), respectively), compared to those who were unemployed. However, professional occupation was associated with higher odds of being infected among both young men and women. Living in neighbourhoods with a high proportion of employed residents was associated with lower odds of HIV infection among young women and men. Conversely, living in a neighbourhood with high wealth and education status was associated with higher odds of HIV infection (Table 2).

**Table 1:** HIV prevalence and age-adjusted odds ratios by background characteristics among young people 15–24 years, 2013–14 and 2018, Zambia.

Independent Variables	2013–14				2018			
	Number	HIV %	AOR (95% CI)	P-value	Number	HIV %	AOR (95% CI)	P-value
**Individual Variables**								
**Residence**								
Rural	5897	2.4	1		5539	2.6	1	
Urban	5674	6.6	3.21 (2.59–3.99)	**< 0.001**	4615	5.3	2.02 (1.57–2.60)	**< 0.001**
**Age** ^1^								
15–19	6519	2.6	1		5557	1.9	1	
20–24	5052	6.8	2.82 (2.34–3.40)	**< 0.001**	4598	6.1	3.20 (2.52–4.07)	**< 0.001**
**Marital Status**								
Never married	8585	3.5	1		7648	3.1	1	
Married	2657	6.2	1.37 (1.10–1.70)	0.005	2245	5.2	1.27 (0.97–1.66)	0.088
Formerly married	329	15.7	3.25 (2.26–4.67)	**< 0.001**	262	13.9	4.03 (2.66–6.10)	**< 0.001**
**Education**								
No education	279	7.1	1		327	2.9	1	
Primary	4181	4.1	0.88 (0.51–1.52)	0.637	3923	3.6	1.01 (0.53–1.93)	0.969
Secondary	6779	4.5	0.83 (0.48–1.43)	0.504	5626	4.0	1.03 (0.55–1.96)	0.917
Higher than secondary	324	5.3	0.57 (0.27–1.22)	0.148	278	4.4	0.71 (0.28–1.79)	0.465
**Wealth**								
Low	3369	2.5	1		2813	2.3	1	
Medium	3566	5.0	2.22 (1.70–2.90)	**< 0.001**	3323	3.9	1.60 (1,18–2.17)	**0.002**
High	4636	5.5	2.38 (1.82–3.11)	**< 0.001**	4019	4.8	1.80 (1.32–2.46)	**< 0.001**
**Employment**								
Not employed	6487	4.7	1		5696	3.6	1	
Employed	5084	4.2	0.67 (0.55–0.80)	**< 0.001**	4458	4.0	0.0.75(0.60–0.95)	**0.016**
**Occupation**								
Unemployed	6497	4.7	1		5696	3.6	1	
Professional	1359	9.5	1.37 (1.11–1.75)	0.004	1089	6.5	1.10 (0.80–1.52)	0.564
Agricultural	2950	1.8	0.31 (0.23–0.41)	**< 0.001**	1587	2.4	0.51 (0.35–0.74)	**< 0.001**
Manual	547	4.2	0.54 (0.33–0.87)	**0.012**	1768	4.0	0.76 (0.55–1.04)	0.084
**Neighbourhood Variables**								
**Neighbourhood education**								
Low	3421	1.9	1		2917	2.1	1	
Medium	3974	4.7	2.46 (1.82–3.32)	**< 0.001**	3277	3.5	1.73 (1.23–2.43)	**0.001**
High	4176	6.3	3.60 (2.69–4.82)	**< 0.001**	3960	5.4	2.42 (1.75–3.36)	**< 0.001**
**Neighbourhood wealth**								
Low	3471	2.0	1		2914	1.8	1	
Medium	3326	4.4	2.38 (1.77–3.22)	**< 0.001**	3060	3.7	2.28 (1.62–3.22)	**< 0.001**
High	4774	6.2	3.58 (2.64–4.72)	**< 0.001**	4181	5.3	2.75 (1.96–3.87)	**< 0.001**
**Neighbourhood employment**								
Low	4815	4.2	1		3646	3.9	1	
Medium	3624	6.0	1.23 (0.97–1.57)	**0.085**	3268	4.2	1.13 (0.83–1.53)	0.433
High	3132	3.0	0.58 (0.44–0.77)	**< 0.001**	3241	3.3	0.84 (0.61–1.15)	0.276
Total	11571	4.5			10154	3.8		

^1^ Age categories are not age adjusted with a continuous age variable.

**Table 2:** HIV prevalence and age-adjusted odds ratios by background characteristics and sex among young people 15–24 years, 2013–14 and 2018, Zambia.

Independent Variables	2013-14								2018							
	Male				Female				Male				Female			
	Number	HIV%	AOR	CI	Number	HIV%	AOR	CI	Number	HIV%	AOR	CI	Number	HIV%	AOR	CI
**Residence**																
Urban	2836	1.1	1		3061	3.6	1		2636	1.0	1		2903	4.0	1	
Rural	2717	3.9	3.66	(2.34–5.73)***	2957	9.1	3.09	(2.44–3.92)***	2126	2.7	2.29	(1.36–3.86)***	2489	7.5	1.93	(1.45–2.58)***
**Age^2^**																
15–19	3246	1.8	1		3273	3.5	1		2738	1.2	1		2818	2.6	1	
20–24	2307	3.5	1.96	(1.34–2.86)***	2745	9.6	3.05	(2.46–3.80)***	2023	2.5	1.83	(1.12–2.98)**	2574	8.9	3.64	(2.74–4.83)***
**Marital Status**																
Never Married	4947	2.2	1		3638	5.2	1		4253	1.7	1		3395	4.8	1	
Married/Co-Habiting	549	4.3	1.38	(0.78–2.43)	2108	6.7	0.80	(0.63–1.03)*	478	1.4	0.84	(0.36–1.97)	1767	6.2	0.82	(0.61–1.11)
Formerly Married	57	6.4	2.23	(0.65–7.65)	272	17.7	1.94	(1.32–2.86)***	31	16.9	12.27	(3.82–39.40)***	231	13.4	2.05	(1.30–3.24)***
**Education**																
No Education	93	2.9	1		185	9.2	1		139	2.4	1		188	3.3	1	
Primary	1937	2.5	0.61	(0.18–2.08)	2244	5.5	1.08	(0.59–1.98)	1806	1.7	0.91	(0.21–4.05)	2117	5.1	1.13	(0.54–2.33)
Secondary	3338	2.3	0.60	(0.18–1.99)	3441	6.7	1.22	(0.67–2.22)	2679	1.6	0.73	(0.17–3.23)	2947	6.2	1.36	(0.66–2.80)
Tertiary/Higher	181	5.2	0.87	(0.20–3.72)	143	5.4	0.69	(0.26–1.72)	138	5.1	2.16	(0.38–12.35)	140	3.6	0.46	(0.13–1.60)
**Wealth**																
Low	1519	1.4	1		1850	3.5	1		1207	1.0	1		1606	3.3	1	
Medium	1779	2.3	1.89	(1.09–3.29)**	1787	7.6	2.53	(1.88–3.41)***	1685	1.1	1.22	(0.5960–2.52)	1638	6.9	1.86	(1.32–2.62)***
High	2255	3.3	2.33	(1.36–3.99)***	2381	7.5	2.70	(2.00–3.65)***	1869	2.9	2.51	(1.29–4.88)***	2149	1.76	1.79	(1.25–2.54)***
**Employment**																
Not Employed	2373	2.6	1		4114	5.9	1		2063	1.5	1		3633	4.8	1	
Employed	3180	2.4	0.64	(0.43–0.95)**	1904	7.2	0.94	(0.76–1.18)	2698	2.0	0.84	(0.49–1.44)	1760	7.2	1.08	(0.83–1.41)
**Occupation**																
Unemployed	2379	2.6	1		4117	5.9	1		2063	1.5	1		3633	4.8	1	
Professional	607	5.1	1.44	(0.86–2.41)	752	13.1	1.58	(1.22–2.03)***	451	2.9	1.09	(0.47–2.55)	638	9.1	1.20	(0.84–1.72)
Agriculture	1 871	1.0	0.27	(0.15–0.49)***	1079	3.2	0.45	(0.32–0.63)***	856	1.3	0.55	(0.25–1.22)	731	3.8	0.67	(0.44–1.03)*
Manual/Domestic	520	4.3	1.18	(0.66–2.10)	27	2.4	0.80	(0.18–3.52)	1390	2.1	0.98	(0.53–1.79)	378	0.9	1.74	(1.15–2.65)***
**Cluster Level**																
**Education**																
Low	1646	0.5	1		1771	3.3	1		1373	0.7	1		1545	3.2	1	
Medium	1894	3.0	4.05	(2.01–8.15)***	2081	6.4	2.19	(1.58–3.03)***	1548	1.7	2.15	(1.02–4.55)**	1729	5.0	1.70	(1.16–2.49)***
High	2013	3.6	5.70	(2.89–11.25)***	2163	8.7	3.22	(2.36–4.40)***	1841	2.6	3.00	(1.45–6.23)***	2119	7.8	2.31	(1.60–3.34)***
**Wealth**																
Low	1636	0.6	1		1835	3.3	1		1363	0.8	1		1561	2.7	1	
Medium	1612	2.3	4.04	(1.94–8.40)***	1714	6.5	2.18	(1.58–3.00)***	14785	1.3	1.70	(0.81–3.58)	1583	5.9	2.52	(1.71–3.73)***
High	2305	3.9	6.51	(3.21–13.22)***	2469	8.7	3.19	(2.34–4.35)***	1921	2.8	3.05	(1.51–6.17)***	2259	7.4	2.66	(1.81–3.92)***
**Employment**																
Low	2297	2.7	1		2519	5.6	1		1655	1.7	1		1991	5.8	1	
Medium	1746	3.1	0.82	(0.53–1.27)	1878	8.7	1.37	(1.06–1.77)**	1496	1.9	1.06	(0.56–2.03)	1772	6.1	1.17	(0.83–1.65)
High	1510	1.3	0.47	(0.28–0.79)***	1622	4.7	0.63	(0.46–0.85)***	1611	1.7	1.04	(0.55–1.98)	1630	4.8	0.84	(0.58–1.21)
	5553	2.5			6018	6.3			4762	1.8			5393	5.6		

Figures with asterix are significant at the following

* p<0.10,

**p<0.05,

*** p<0.01.

^1^Age categories are not age adjusted with a continuous age variable.

The intercept-only model showed that the overall HIV prevalence varied across neighbourhoods with a neighbourhood difference of 14% in 2013–14 (results not shown) (see S3 Table). In Model 2, the addition of individual-level variables to the intercept-only model explained more of the variance than the addition of neighbourhood variables (it explained a variance of 62% and a neighbourhood difference of 7% versus 9% and 8%, respectively). The inclusion of both individual and neighbourhood variables in Model 4 explained 62.4% of the variance in HIV prevalence, and the likelihood-ratio test showed that the fit of the model significantly improved compared to the intercept model. The following variables were associated with higher odds of being HIV positive in Model 4: age, urban residence, formerly married, medium wealth and residing in neighbourhoods with high educational attainment and medium-level employment. Educational attainment at the individual level and employment were significantly associated with lower odds of being infected (results not shown) (see S3 Table).

For 2018, the intercept-only model showed 13% variation in HIV prevalence across neighbourhoods (results not shown) (see S3 Table). In Model 2, the addition of individual-level variables to the intercept-only model explained 58% of the variance and a neighbourhood difference of 11%. The inclusion of both individual and neighbourhood variables in Model 4 explained 59% of the variance in HIV prevalence, and the likelihood-ratio test showed that the fit of the model significantly improved compared to the intercept model. The following variables were associated with higher odds of being HIV positive in Model 4: age, urban residence, married or formerly married, and residing in neighbourhoods with medium-level wealth. Employment was significantly associated with lower odds of being infected (results not shown) (see S3 Table).

Among young males, the intercept-only model shows that HIV prevalence varied by 13% across neighbourhoods in 2013–14. The inclusion of both individual and neighbourhood variables explained 59.5% of the variance in HIV prevalence, with a neighbourhood difference of 2%. The likelihood-ratio test showed that the inclusion of both individual and neighbourhood variables to the intercept model improved the fit of the model. Factors associated with higher HIV infection were age, urban residence and high neighbourhood education and wealth. Individual educational attainment and employment were associated with reduced odds of HIV infection (Table 3).

**Table 3:** Multilevel logistic regression models for HIV prevalence among young men aged 15–24 years in Zambia, 2013–14 and 2018.

	2013–14							2018						
	Model 1	Model 2		Model 3		Model 4		Model 1	Model 2		Model 3		Model 4	
		AOR	95% CI	AOR	95% CI	AOR	95% CI		AOR	95% CI	AOR	95% CI	AOR	95% CI
**Fixed Effects**														
**Independent Variables**														
**Age**		1.20	(1.10–1.31)***			1.20	(1.10–1.31)***		1.13	(1.01–1.26)**			1.14	(1.02–1.27)**
**Education**		0.92	(0.85–0.99)**			0.89	(0.82–0.97)**		0.92	(0.82–1.02)*			0.90	(0.80–1.00)**
**Residence**														
Rural		1				1			1				1	
Urban		4.28	(2.42–7.58)***			2.23	(1.22–4.08)***		1.69	(0.83–3.43)			1.45	(0.64–3.25)
**Marital Status**														
Never Married		1				1			1				1	
Married/Co-Habiting		1.58	(0.85–2.93)			1.63	(0.88–3.02)		1.10	(0.44–2.75)			1.11	(0.44–2.78)
Formerly Married		1.91	(0.55–6.63)			1.91	(0.55–6.69)		13.29	(3.99–44.25)***			13.12	(3.93–43.75)***
**Wealth**														
Low		1				1			1				1	
Medium		1.16	(1.15–2.11)***			0.78	(0.40–1.50)		1.30	(0.59–2.88)			0.99	(0.42–2.37)
High		0.98	(0.91–1.91)			0.51	(0.23–1.12)		2.49	(0.94–6.57)*			1.75	(0.58–5.27)
**Employment**														
Not Employed		1				1			1				1	
Employed		0.59	(0.38–0.91)**			0.59	(0.37–0.94)**		0.87	(0.49–1. 55)			0.83	(0.46–1.48)
**Neighbourhood Variables**														
**Education**														
Low				1		1					1		1	
Medium				2.27	(1.03–5.03)**	2.53	(1.12–5.74)**				1.75	(0.70–4.43)	2.09	(0.79–5.56)
High				2.19	(0.86–5.54)	2.70	(1.03–7.08)**				1.50	(0.45–5.06)	1.97	(0.55–7.09)
**Wealth**														
Low				1		1					1		1	
Medium				2.67	(1.16–6.15)**	2.41	(0.96 1.55)*				1.29	(0.52–3.23)	1.14	(0.41–3.16)
High				3.93	(1.48–10.46)***	3.62	(1.15–11.35) **				2.57	(0.79–8.42)	1.31	(0.30–5.77)
**Employment**														
Low				1		1					1		1	
Medium				0.85	(0.55–1.32)	0.89	(0.58–1.38)				1.11	(0.59–2.09)	1.11	(0.58–2.12)
High				0.90	(0.51–1.59)	0.98	(0.54–1.76)				1.43	(0.874–2.75)	1.44	(0.82–2.84)
**Unexplained neighbourhood-level variance (SE)**	0.512	0.268	(0.32)	0.192	(0.31)	0.067	(0.30)	0.84	0.69	(0.48)**	0.65	(0.46)***	0.67	(0.47)***
**Model Statistics**														
Explained variance (R-Squared)		0.564		0.18		0.595			0.4157		0.07		0.443	
Intraclass correlation (ICC rho)	0.13	0.040		0.05		0.02		0.204	0.17		0.17		0.17	
Log Likelihood	-551.60	-516.70		-528.60		-506.38		-353.01	-334.88		-345.92		-332.62	
Likelihood-ratio test*		69.81***	<0.01	46.35***	<0.01	90.44***	<0.01		36.2***	<0.01	14.17**	0.028	40.78***	<0.01

Figures with asterix are significant at the following * p<0.10, **p<0.05, **** p<0.01; likelihood-ratio test*—Tests whether adding individual and neighbourhood variables to the null model (model 1) significantly improves the fit of the model (P<0.05); Abbreviations R^2^- Explained variance; ICC—Inter-class correlation; AOR: age—adjusted Odds Ratio and CI: Confidence Interval.

The intercept-only model in 2018 shows that HIV prevalence varied by 20% across neighbourhoods. The inclusion of both individual and neighbourhood variables explained 44.3% of the variance in HIV prevalence, with a neighbourhood difference of 17%. The likelihood-ratio test showed that the inclusion of both individual and neighbourhood variables to the intercept model improved the fit of the model. Factors associated with higher HIV infection were age and being formerly married. Individual educational attainment was associated with reduced odds of HIV infection (Table 3).

Model 1 in 2013–14 showed that HIV prevalence among females varied across neighbourhoods, with a statistically significant neighbourhood difference of 11%. The addition of both individual and neighbourhood variables in Model 4 explained 65.7% of the variance, with a neighbourhood difference of 4%. The likelihood-ratio test showed that the inclusion of both individual and neighbourhood variables to the intercept model improved the fit of the model. Residing in urban areas, older age, being formerly married, wealth and residing in neighbourhoods with medium-level employment were associated with higher odds of being HIV positive. Educational attainment at the individual level was associated with lower odds of being infected. High neighbourhood education was associated with increased odds of HIV infection (Table 4).

**Table 4:** Multilevel logistic regression models for HIV prevalence among young women aged 15–24 years in Zambia, 2013–14 and 2018.

	2013–14							2018						
	Model 1	Model 2		Model 3		Model 4		Model 1	Model 2		Model 3		Model 4	
		AOR	95% CI	AOR	95% CI	AOR	95% CI		AOR	95% CI	AOR	95% CI	AOR	95% CI
**Fixed Effects**														
**Independent Variables**														
**Age**		1.27	(1.21–1.33)***			1.27	(1.21–1.33)***		1.27	(1.20–1.34)***			1.27	(1.20–1.34)***
**Education**		0.96	(0.92–0.99)**			0.96	(0.92–0.98)***		0.98	(0.93–1.03)			0.96	(0.92–1.01)
**Residence**														
Rural		1				1			1				1	
Urban		2.71	(2.01–3.64)***			2.24	(1.58–3.17)***		1.83	(1.26–2.64)***			1.50	(0.98–2.29)*
**Marital Status**														
Never Married		1				1			1				1	
Married/Co-Habiting		0.78	(0.60–1.01)*			0.77	(0.59–1.00)**		0.84	(0.60–1.16)			0.84	(0.60–1.17)
Formerly Married		1.75	(1.17–2.63)***			1.69	(1.13–2.54)***		1.92	(1.18–3.13)***			1.89	(0.54–3.08)***
**Wealth**														
Low		1				1			1				1	
Medium		1.74	(1.23–2.46)***			1.61	(1.11–2.33)***		1.54	(1.05–2.26)**			1.18	(0.79–1.17)
High		1.48	(0.98–2.23)			1.26	(0.79–1.99)		1.20	(0.74–1.97)			0.92	(0.58–1.58)
**Employment**														
Not Employed		1				1			1				1	
Employed		1.05	(0.83–1.32))			1.06	(0.83–1.33)		1.02	(0.78–1.35)			1.04	(0.78–1.37)
**Neighbourhood Variables**														
**Education**														
Low				1		1					1		1	
Medium				1.59	(1.08–2.32)**	1.57	(1.06–2.33)**				1.06	(0.67–1.69)	1.16	(0.71–1.88)
High				1.90	(1.19–3.06)***	1.84	(1.12–3.01)**				1.42	(0.78–2.61)	1.48	(0.79–2.80)
**Wealth**														
Low				1		1					1		1	
Medium				1.49	(1.01–2.19)**	0.87	(0.55–1.37)				2.25	(1.39–3.63)***	1.98	(1.17–3.35)***
High				1.88	(1.16–3.05)***	0.92	(0.51–1.65)				2.01	(1.06–3.81)**	1.58	(0.72–1.56)
**Employment**														
Low				1		1					1		1	
Medium				1.51	(1.18–1.94)***	1.40	(1.09–1.80)**				1.21	(0.87–1.67)	1.15	(0.82–1.60)
High				1.10	(0.79–1.52)	1.02	(0.73–1.44)				1.12	(078–1.52)	1.06	(0.72–1.56)
**Unexplained neighbourhood-level variance (SE)**	0.400	0.173	(0.10)**	0.210	(0.10)***	0.130	(0.09)***	0.555	0.47	(0.17)***	0.39	(0.16)***	0.40	(0.17)***
**Model Statistics**														
Explained variance (R-Squared)		0.652		0.08		0.657			0.640		0.054		0.646	
Intraclass correlation (ICC rho)	0.11	0.050		0.06		0.04		0.14	0.12		0.11		0.11	
Log Likelihood	-1551.67	-1421.33		-1512.56		-1413.12		-1088.16	-1016.29		-1070.98		-1009.14	
Likelihood-ratio test*		260.68***	<0.01	79.27***	<0.01	277.03***	<0.01		143.75***	<0.01	34.37***	<0.01	158.05***	<0.01

Figures with asterix are significant at the following * p<0.10, **p<0.05, **** p<0.01; likelihood-ratio test*—Tests whether adding individual and neighbourhood variables to the null model (model 1) significantly improves the fit of the model (P<0.05); Abbreviations R^2^- Explained variance; ICC—Inter-class correlation; AOR: age—adjusted Odds Ratio and CI: Confidence Interval.

For 2018 the intercept-only model showed a higher variation in HIV prevalence across neighbourhoods of 14%. The addition of both individual and neighbourhood variables in Model 4 explained 64.6% of the variance, with a neighbourhood difference of 11%. The likelihood-ratio test showed that the inclusion of both individual and neighbourhood variables to the intercept model improved the fit of the model. Older age, being formerly married and residing in neighbourhoods with medium-level wealth were associated with higher odds of being HIV positive among females (Table 4).

## Discussion

Our analysis indicates that both individual and neighbourhood socioeconomic factors influence the likelihood of young people being infected with HIV in Zambia. At the individual level, older age and urban residence were found to be associated with increased odds of infection for both young men and women. Formerly married women were also at increased odds of HIV infection in both 2013–14 and 2018, while formerly married young men were associated with increased HIV risk in 2018. Education was associated with a reduced risk of HIV infection for both sexes. Young employed men were also at reduced risk of infection. In the full multivariate model only neighbourhood education was associated with higher odds of HIV infection for both young men and women in 2013. Living in a neighbourhood of medium wealth was associated with higher odds of HIV infection for young women in 2018.

Comparison of the different multilevel models indicate that inclusion of individual factors explained more of the variance compared to neighbourhood-level factors, suggesting that individual level factors played a more important role in young people’s risk of HIV infection. The finding that the variation in HIV infection attributed to neighbourhoods was lower in the models with individual factors particularly in 2013–14, strengthens this interpretation.

Education was found to reduce the risk of infection in young people. An additional year of educational attainment reduced the odds of being infected by 11% and 10% for young men and 4% for young women in 2013–14 and 2018, respectively. However, the strength of association reduced in 2018 among young women. As mentioned earlier, some studies have documented similar findings [10,15,16]. A systematic review assessing the association between education and HIV by Hargreaves et al. found that studies in sub-Saharan Africa done from 1996 onwards showed a lower risk of infection among the most educated [16]. We also found that in the multivariate analysis, after adjusting for neighbourhood factors, the protective effect of individual education was maintained. It may reflect that young people with high education may feel more empowered to make decisions that can protect them from HIV infection, contrary to the less-educated young people. Educated persons also tend to be early adopters of new practices; they internalize relevant information and translate this knowledge into behavioural change [28].

It has been postulated that the spread of education changes the community environment and promotes socially acceptable behaviour, i.e., delayed sexual debut, limiting sexual partners, etc. [28]. Therefore, people living in neighbourhoods with high educational attainment could be expected to be more likely to adopt health-promoting behaviours. Thus, it is logical to expect that neighbourhood-level educational attainment might also be protective. To the contrary, our study found that neighbourhood-level educational attainment was associated with increased risk of HIV infection. These findings contradict the findings from the earlier studies done in selected areas of Zambia, which found that neighbourhood education was associated with a reduced risk of HIV infection. It could be that the two studies were not representative of the whole population or that the contextual factors affecting risk are not static and may change in their effect as the HIV epidemic evolves.

Living in a neighbourhood with medium-level employment and wealth was found to be a predictor of higher odds of HIV infection for young women in 2013 and 2018, respectively. Neighbourhoods with a medium proportion of persons employed may be more heterogeneous in terms of the socioeconomic status of the inhabitants and have more disposable resources available than neighbourhoods with higher levels of unemployment, thereby negatively affecting young peoples’ sexual behaviour. We postulate that those with more resources in communities where others are unemployed may be more likely to engage in particular risky lifestyles, such as higher rates of partner change and multiple sexual partners, because of greater autonomy and spatial mobility. The statistical evidence of a positive association between neighbourhood employment and high HIV prevalence is surprising and needs further investigation. Our findings indicate that the associations with socioeconomic status may change continuously over time, and that there is a need for more nuanced terms to describe the different stages of the HIV epidemic. Further, the findings may also suggest that the association between neighbourhood-level socio-economic factors and HIV prevalence is complex. The correlation between neighbourhood wealth and education suggests that the two SEP indicators capture partially similar underlying contextual aspects of the neighbourhood.

Our study has some limitations. As in any cross-sectional study, the exposures and outcome were measured simultaneously in this given population. This limits our ability to draw causal inferences about the associations found. However, since the study was limited to young people, HIV infection was likely to be recent. The assumption is that they have recently become sexually active and are not likely to be greatly affected by mortality and treatment, and only a small proportion would have been infected by their mother. According to the Zambia population-based HIV impact assessment (ZAMPHIA) survey, about 1% of children (0–14 years) were HIV positive, most likely infected by their mothers [29]. Some important factors may have biased the associations with neighbourhood characteristics in our study, such as using enumeration areas as a proxy of neighbourhoods. These clusters are not necessarily representative of naturally occurring neighbourhoods where individuals reside or residential areas. Studies have, however, found them to be small enough to be a useful proxy [30]. A high proportion of the total variance was not explained by the neighbourhood variables used. This could be partly because there was no information on more specific community-level characteristics that have been associated with HIV prevalence in other studies, such as the availability of markets, health facilities, bars, and proximity to trading areas and major roads. Mobility, which has been linked to the risk of HIV infection in numerous studies, was also not measured in our study, and thus we do not know how long respondents had lived in the neighbourhood where they were interviewed. Another limitation is that the only neighbourhood factors that are measured are aggregate individual responses within each enumeration area which is the proxy neighbourhood.

## Conclusion

This study using nationally representative data indicates that individual socioeconomic factors are more strongly associated with HIV infection than neigbourhood factors among young people in Zambia. Individual-level education remains an important socioeconomic factor associated with reduced odds of HIV infection. This suggests that the HIV response in Zambia should still focus on individual level prevention strategies. Further, contrasting associations between HIV Infection risk and neighbourhood-level education and individual-level education suggests a complex relationship between the two levels that requires further investigation.

## Supporting information

S1 Table Descriptive statistics of young people aged 15–24 years stratified by sex, 2013–14 ZDHS. (DOCX)

S2 Table Descriptive statistics of young people aged 15–24 years stratified by sex, 2018 ZDHS. (DOCX)

S3 Table Multilevel logistic regression models for HIV prevalence among young people aged 15–24 years in Zambia, both sexes, 2013–14 and 2018. (DOCX)

S4 Table Multilevel logistic regression Model 5 (including interaction terms) for HIV prevalence among young people aged 15–24 years in Zambia, both sexes, 2013–14 and 2018. (DOCX)
