# Supplementary material for: Examining the association between HIV prevalence and socioeconomic factors among young people in Zambia: Do neighbourhood contextual effects play a role?
Source: PLoS One. 2022 Jun 8;17(6):e0268983. doi: 10.1371/journal.pone.0268983 (PMC9176771; doi:10.1371/journal.pone.0268983)
Supplement: S1 Table — (DOCX) [file pone.0268983.s001.docx]

|  | **Total** | |  | **Male** | |  | **Female** | |
| --- | --- | --- | --- | --- | --- | --- | --- | --- |
| **Independent Variables** | **Number** | **% Distribution** |  | **Number** | **% Distribution** |  | **Number** | **% Distribution** |
| **Residence** |  |  |  |  |  |  |  |  |
| Rural | 5897 | 51.0 |  | 2836 | 51.1 |  | 3061 | 50.9 |
| Urban | 5674 | 49.0 |  | 2717 | 48.9 |  | 2957 | 49.1 |
| **Age** |  |  |  |  |  |  |  |  |
| 15–19 | 6519 | 56.3 |  | 3246 | 58.4 |  | 3273 | 54.4 |
| 20–24 | 5052 | 43.7 |  | 2307 | 41.6 |  | 2745 | 45.6 |
| **Sex** |  |  |  |  |  |  |  |  |
| Male | 5553 | 48.0 |  | 5553 | - |  | 6018 | - |
| Female | 6018 | 52.0 |  |  |  |  |  |  |
| **Marital Status** |  |  |  |  |  |  |  |  |
| Never married | 8585 | 74.2 |  | 4947 | 89.1 |  | 3638 | 60.5 |
| Married | 2657 | 23.0 |  | 549 | 9.9 |  | 2108 | 35.0 |
| Formerly married | 329 | 2.8 |  | 57 | 1.0 |  | 272 | 4.5 |
| **Education** |  |  |  |  |  |  |  |  |
| No education | 279 | 2.4 |  | 93 | 1.7 |  | 185 | 3.1 |
| Primary | 4181 | 36.1 |  | 1937 | 34.9 |  | 2244 | 37.3 |
| Secondary | 6779 | 58.6 |  | 3338 | 60.1 |  | 3441 | 57.2 |
| Higher than secondary | 324 | 2.8 |  | 181 | 3.3 |  | 143 | 2.4 |

**SI Table: Descriptive Statistics of young people aged 15-24 years stratified by sex, 2013-14 ZDHS**
