# Supplementary material for: Examining the association between HIV prevalence and socioeconomic factors among young people in Zambia: Do neighbourhood contextual effects play a role?
Source: PLoS One. 2022 Jun 8;17(6):e0268983. doi: 10.1371/journal.pone.0268983 (PMC9176771; doi:10.1371/journal.pone.0268983)
Supplement: S2 Table — (DOCX) [file pone.0268983.s002.docx]

**S2 Table: Descriptive Statistics of young people aged 15-24 years stratified by sex, 2018 ZDHS**

|  | **Total** | |  | **Male** | |  | **Female** | |
| --- | --- | --- | --- | --- | --- | --- | --- | --- |
| **Independent Variables** | **Number** | **% Distribution** |  | **Number** | **% Distribution** |  | **Number** | **% Distribution** |
| **Residence** |  |  |  |  |  |  |  |  |
| Rural | 5539 | 54.6 |  | 2636 | 55.4 |  | 2903 | 53.8 |
| Urban | 4615 | 45.4 |  | 2126 | 44.6 |  | 2489 | 46.2 |
| **Age** |  |  |  |  |  |  |  |  |
| 15–19 | 5557 | 54.7 |  | 2738 | 57.5 |  | 2818 | 52.3 |
| 20–24 | 4598 | 45.3 |  | 2023 | 42.5 |  | 2574 | 47.7 |
| **Sex** |  |  |  |  |  |  |  |  |
| Male | 4762 | 46.9 |  | 4762 | - |  | 5393 | - |
| Female | 5393 | 53.1 |  |  |  |  |  | - |
| **Marital Status** |  |  |  |  |  |  |  |  |
| Never married | 7648 | 75.3 |  | 4253 | 89.3 |  | 3395 | 63.0 |
| Married | 2245 | 22.1 |  | 478 | 10.0 |  | 1767 | 32.8 |
| Formerly married | 262 | 2.6 |  | 31 | 0.7 |  | 231 | 4.3 |
| **Education** |  |  |  |  |  |  |  |  |
| No education | 327 | 3.2 |  | 139 | 2.9 |  | 188 | 3.5 |
| Primary | 3923 | 38.6 |  | 1806 | 37.9 |  | 2117 | 39.3 |
| Secondary | 5626 | 55.4 |  | 2679 | 56.3 |  | 2947 | 54.7 |
| Higher than secondary | 278 | 2.7 |  | 138 | 2.9 |  | 140 | 2.6 |
