# Supplementary material for: Examining the association between HIV prevalence and socioeconomic factors among young people in Zambia: Do neighbourhood contextual effects play a role?
Source: PLoS One. 2022 Jun 8;17(6):e0268983. doi: 10.1371/journal.pone.0268983 (PMC9176771; doi:10.1371/journal.pone.0268983)
Supplement: S3 Table — (DOCX) [file pone.0268983.s003.docx]

**S3 Table: Multilevel logistic regression models for HIV prevalence among young people aged 15–24 years in Zambia, both sexes, 2013–14 and 2018.**

|  | **2013-14** | | | | | | | | | |  | **2018** | | | | | | | | | |
| --- | --- | --- | --- | --- | --- | --- | --- | --- | --- | --- | --- | --- | --- | --- | --- | --- | --- | --- | --- | --- | --- |
|  | **Model 1** |  | **Model 2** | |  | **Model 3** | |  | **Model 4** | |  | **Model 1** |  | **Model 2** | |  | **Model 3** | |  | **Model 4** | |
|  |  |  | **AOR** | **95% CI** |  | **AOR** | **95% CI** |  | **AOR** | **95% CI** |  |  |  | **AOR** | **95% CI** |  | **AOR** | **95% CI** |  | **AOR** | **95% CI** |
| **Fixed Effects** |  |  |  |  |  |  |  |  |  |  |  |  |  |  |  |  |  |  |  |  |  |
| **Independent Variables** |  |  |  |  |  |  |  |  |  |  |  |  |  |  |  |  |  |  |  |  |  |
| **Age** |  |  | 1.24 | (1.19 - 1.29)*** | |  |  |  | 1.24 | (1.19 - 1.29)*** | |  |  | 1.22 | (1.16 - 1.28)*** | |  |  |  | 1.22 | (1.16 - 1.28)*** |
| **Education** |  |  | 0.94 | (0.91 - 0.98)*** | |  |  |  | 0.93 | (0.89 - 0.96)*** | |  |  | 0.99 | (0.95 - 1.04) |  |  |  |  | 0.97 | (0.93 - 1.02) |
| **Residence** |  |  |  |  |  |  |  |  |  |  |  |  |  |  |  |  |  |  |  |  |  |
| Rural |  |  | 1 |  |  |  |  |  | 1 |  |  |  |  | 1 |  |  |  |  |  | 1 |  |
| Urban |  |  | 3.11 | (2.37 - 4.08)*** | |  |  |  | 2.28 | (1.66 - 3.12)*** | |  |  | 1.95 | (1.40 - 2.70)*** | |  |  |  | 1.59 | (1.09 - 2.13)** |
| **Marital Status** |  |  |  |  |  |  |  |  |  |  |  |  |  |  |  |  |  |  |  |  |  |
| Never Married |  |  | 1 |  |  |  |  |  | 1 |  |  |  |  | 1 |  |  |  |  |  | 1 |  |
| Married/Co-Habiting |  |  | 1.26 | (1.00 - 1.60)** | |  |  |  | 1.25 | (0.98 - 1.58)* |  |  |  | 1.35 | (1. 01 - 1.80)** | |  |  |  | 1. 35 | (1.01 - 1.81)** |
| Formerly Married |  |  | 2.80 | (1.92 - 4.07)*** | |  |  |  | 2.72 | (1.86 - 3.96)*** | |  |  | 4.08 | (2.64 - 6.30)*** | |  |  |  | 3.99 | (2.58 - 6.16)*** |
| **Wealth** |  |  |  |  |  |  |  |  |  |  |  |  |  |  |  |  |  |  |  |  |  |
| Low |  |  | 1 |  |  |  |  |  | 1 |  |  |  |  | 1 |  |  |  |  |  | 1 |  |
| Medium |  |  | 1.55 | (1.15 - 2.11)*** | |  |  |  | 1.36 | (0.98 - 1.87)* | |  |  | 1.39 | (0.99 - 1.95)* |  |  |  |  | 1.07 | (0.75 - 1.55) |
| High |  |  | 1.33 | (0.91 - 1.91) | |  |  |  | 1.03 | (0.69 - 1.53) |  |  |  | 1.26 | (0.82 - 1.95) |  |  |  |  | 0.94 | (0.58 - 1.52) |
| **Employment** |  |  |  |  |  |  |  |  |  |  |  |  |  |  |  |  |  |  |  |  |  |
| Not Employed |  |  | 1 |  |  |  |  |  | 1 |  |  |  |  | 1 |  |  |  |  |  | 1 |  |
| Employed |  |  | 0.69 | (0.57 - 0.84)*** | |  |  |  | 0.69 | (0.56 - 0.85)*** | |  |  | 0.74 | (0.58 - 0.94)*** | |  |  |  | 0.74 | (0.58 - 0.94)*** |
| **Neighbourhood Variables** |  |  |  |  |  |  |  |  |  |  |  |  |  |  |  |  |  |  |  |  |  |
| **Education** |  |  |  |  |  |  |  |  |  |  |  |  |  |  |  |  |  |  |  |  |  |
| Low |  |  |  |  |  | 1 |  |  | 1 |  |  |  |  |  |  |  | 1 |  |  | 1 |  |
| Medium |  |  |  |  |  | 1.72 | (1.21 - 2.45)*** | | 1.80 | (1.25 - 2.59)*** | |  |  |  |  |  | 1.16 | (0.77 - 1.77) |  | 1.27 | (0.82 - 1.95) |
| High |  |  |  |  |  | 2.04 | (1.32 - 3,17)*** | | 2.22 | (1.41 - 3.51)*** | |  |  |  |  |  | 1.44 | (0.83 - 2.48) |  | 1.60 | (0.90 - 2.83) |
| **Wealth** |  |  |  |  |  |  |  |  |  |  |  |  |  |  |  |  |  |  |  |  |  |
| Low |  |  |  |  |  | 1 |  |  | 1 |  |  |  |  |  |  |  | 1 |  |  | 1 |  |
| Medium |  |  |  |  |  | 1.59 | (1.11 - 2.27)*** | | 1.02 | (0.68 - 1.55) |  |  |  |  |  |  | 1.97 | (1.29 - 3.02)*** | | 1.76 | (1.10 - 2.82)** |
| High |  |  |  |  |  | 2.05 | (1.30 - 3.22)*** | | 1.13 | (0.66 - 1.94) |  |  |  |  |  |  | 2.11 | (1.20 - 3,73)*** | | 1.53 | (0.77 - 3.06) |
| **Employment** |  |  |  |  |  |  |  |  |  |  |  |  |  |  |  |  |  |  |  |  |  |
| Low |  |  |  |  |  | 1 |  |  | 1 |  |  |  |  |  |  |  | 1 |  |  | 1 |  |
| Medium |  |  |  |  |  | 1.32 | (1.05 - 1.67)** | | 1.29 | (1.02 - 1.62)** | |  |  |  |  |  | 1.17 | (0.88 - 1.57) |  | 1.16 | (0.86 - 1.57) |
| High |  |  |  |  |  | 1.01 | (0.75 - 1.36) |  | 1.09 | (0.80 - 1.47) |  |  |  |  |  |  | 1.12 | (0.81 - 1.54) |  | 1.14 | (0.82 - 1.59) |
| **Unexplained neighbourhood-level variance (SE)** | 0.524 |  | 0.268 | (0.09)*** |  | 0.304 | (0.09)*** |  | 0.228 | (0.09)*** |  | 0.484 | (0.13*** | 0.39 | (0.13)*** |  | 0.34 | (0.12)*** |  | 0.34 | (0.13)*** |
| **Model Statistics** |  |  |  |  |  |  |  |  |  |  |  |  |  |  |  |  |  |  |  |  |  |
| Explained variance ( R-Squared) | |  | 0.618 |  |  | 0.09 |  |  | 0.624 |  |  |  |  | 0.578 |  |  | 0.054 |  |  | 0.59 |  |
| Intraclass correlation (ICC rho) | 0.14 |  | 0.070 |  |  | 0.08 |  |  | 0.06 |  |  | 0.128 |  | 0.11 |  |  | 0.09 |  |  | 0.09 |  |
|  |  |  |  |  |  |  |  |  |  |  |  |  |  |  |  |  |  |  |  |  |  |
| Log Likelihood | -2162.28 |  | -1988.56 |  |  | -2110.50 |  |  | -1976.47 |  |  | -1491.59 |  | -1395.63 |  |  | -1470.08 |  |  | -1387.57 |  |
| Likelihood-ratio test* |  |  | 347.44*** | <0.01 |  | 104.99*** | <0.01 |  | 371.62*** | <0.01 |  |  |  | 191.91*** | <0.01 |  | 43.01*** | <0.01 |  | 208.05*** | <0.01 |

Figures with asterix are significant at the following * p<0.10, * *p<0.05, **** p<0.01; **likelihood-ratio test *** - Tests whether adding individual and neighbourhood variables to the null model (model 1) significantly improves the fit of the model (P<0.05); Abbreviations R^2^- Explained variance; ICC – Inter-class correlation; AOR: age – adjusted Odds Ratio and CI: Confidence Interval.
