# Supplementary material for: Examining the association between HIV prevalence and socioeconomic factors among young people in Zambia: Do neighbourhood contextual effects play a role?
Source: PLoS One. 2022 Jun 8;17(6):e0268983. doi: 10.1371/journal.pone.0268983 (PMC9176771; doi:10.1371/journal.pone.0268983)
Supplement: S4 Table — (DOCX) [file pone.0268983.s004.docx]

**S4 Table: Multilevel logistic regression Model 5 (Including Interaction terms) for HIV prevalence among young people aged 15–24 years in Zambia, both sexes, 2013–14 and 2018**

|  | **2013-14** | |  | **2018** | |  |
| --- | --- | --- | --- | --- | --- | --- |
| **Variables** | **AOR 95% CI** | |  | **AOR 95% CI** | |  |
| **Age** | 1.24 | (1.19 – 1.29)*** |  | 1.21 | (1.16 – 1.27)** |  |
| **Education** | 0.96 | (0.90 – 1.02) |  | 0.99 | (0.93 – 1.05) |  |
| **Interactions** |  |  |  |  |  |  |
| **residence*educationyrs** | 0.96 | (0.89 - 1.03) |  | 0.97 | (0.89 – 1.05) |  |
| **Rural/Urban Residence** |  |  |  |  |  |  |
| Rural | 1 |  |  | 1 |  |  |
| Urban | 2.62 | (1.05 – 6.57)** |  | - |  |  |
| **Marital Status** |  |  |  |  |  |  |
| Never Married | 1 |  |  | 1 |  |  |
| Married/Co-Habiting | 1.27 | (1.00 – 1.61)** |  | 1.28 | (0.98 – 1.69)* |  |
| Formerly Married | 2.71 | (1.86 – 3.95)*** |  | 3.92 | (2.59 – 5.92)*** |  |
| **Employment** |  |  |  |  |  |  |
| Not Employed |  |  |  |  |  |  |
| Employed | 0.59 | (0.41 - 0.86)*** |  | 0.78 | (0.57– 1.06) |  |
| **Interactions** |  |  |  |  |  |  |
| Residence*Employed | 1.25 | (0.81 – 1.94) |  | 0.94 | (0.61 – 1.45) |  |
| **Neighbourhood Variables** |  |  |  |  |  |  |
| **Education** |  |  |  |  |  |  |
| Low | 1 |  |  |  |  |  |
| Medium | 1.58 | (1.05 – 2.36)** |  |  |  |  |
| High | 2.82 | (1.44 – 5.52)*** |  |  |  |  |
| **Interactions** |  |  |  |  |  |  |
| Residence*Medium Education | 1.56 | (0.71 – 3.43) |  |  |  |  |
| Residence*High Education | 0.98 | (0.38 - 2.56) |  |  |  |  |
| **Wealth** |  |  |  |  |  |  |
| Low |  |  |  | 1 |  |  |
| Medium |  |  |  | 1.95 | (1.36 – 2.80)*** |  |
| High |  |  |  | 1.87 | (0.91 -3.86)* |  |
| **Interactions** |  |  |  |  |  |  |
| Residence*Medium Education |  |  |  | - |  |  |
| Residence*High Education |  |  |  | - |  |  |
| **Employment** |  |  |  |  |  |  |
| Low | 1 |  |  |  |  |  |
| Medium | 1.24 | (0.75 - 2.07) |  |  |  |  |
| High | 1.33 | (0.82 - 2.16) |  |  |  |  |
| **Interactions** |  |  |  |  |  |  |
| Residence*Medium Employment | 1.06 | (0.60 – 1.88) |  |  |  |  |
| Residence*High Employment | 0.68 | (0.36 - 1.28) |  |  |  |  |
|  |  |  |  |  |  |  |
|  |  |  |  |  |  |  |
| **Unexplained neighbourhood-level variance (SE)** | 0.24 | (0.09)*** |  | 0.29 | (0.11)*** |  |
| **Model Statistics** |  |  |  |  |  |  |
| Explained variance ( R-Squared) | 0.619 |  |  | 0.591 |  |  |
| ICC (rho) | 0.07 |  |  | 0.02 |  |  |

Figures with asterix are significant at the following * p<0.10, * *p<0.05, **** p<0.01; Abbreviations R^2^- Explained variance; ICC – Inter-class correlation; AOR: age – adjusted Odds Ratio and CI: Confidence Interval.
